# Harnessing Ferroptosis to Overcome Drug Resistance in Colorectal Cancer: Promising Therapeutic Approaches

**DOI:** 10.3390/cancers15215209

**Published:** 2023-10-30

**Authors:** Xiaofei Cheng, Feng Zhao, Bingxin Ke, Dong Chen, Fanlong Liu

**Affiliations:** 1 Department of Colorectal Surgery, The First Affiliated Hospital, School of Medicine, Zhejiang University, Hangzhou 310003, China; 1514012@zju.edu.cn (B.K.); cdking@zju.edu.cn (D.C.); 2 Department of Radiation Oncology, The First Affiliated Hospital, School of Medicine, Zhejiang University, Hangzhou 310030, China; zju_zhaofeng@zju.edu.cn

**Keywords:** colorectal cancer (CRC), ferroptosis, drug resistance, treatment strategies, immunotherapy

## Abstract

**Simple Summary:**

In the realm of colorectal cancer treatment, drug resistance is a formidable obstacle. However, a ray of hope emerges in the form of ferroptosis, a unique iron-driven cell death mechanism. This review delves into the role of ferroptosis in colorectal cancer and its implications for drug resistance. Unlike conventional cell death pathways, such as apoptosis and necrosis, ferroptosis offers distinct advantages. We explore current research breakthroughs, including innovative strategies like ferroptosis inducers, iron metabolism and lipid peroxidation interventions, and combination therapies. Additionally, we investigate the potential of immunotherapy in modulating ferroptosis. While targeting ferroptosis presents notable strengths, it comes with challenges in specificity and drug development. Looking ahead, this review underscores the promise of ferroptosis-based therapies in colorectal cancer and emphasizes the need for ongoing research to unlock its full potential, offering renewed hope for colorectal cancer patients.

**Abstract:**

Drug resistance remains a significant challenge in the treatment of colorectal cancer (CRC). In recent years, the emerging field of ferroptosis, a unique form of regulated cell death characterized by iron-dependent lipid peroxidation, has offered new insights and potential therapeutic strategies for overcoming drug resistance in CRC. This review examines the role of ferroptosis in CRC and its impact on drug resistance. It highlights the distinctive features and advantages of ferroptosis compared to other cell death pathways, such as apoptosis and necrosis. Furthermore, the review discusses current research advances in the field, including novel treatment approaches that target ferroptosis. These approaches involve the use of ferroptosis inducers, interventions in iron metabolism and lipid peroxidation, and combination therapies to enhance the efficacy of ferroptosis. The review also explores the potential of immunotherapy in modulating ferroptosis as a therapeutic strategy. Additionally, it evaluates the strengths and limitations of targeting ferroptosis, such as its selectivity, low side effects, and potential to overcome resistance, as well as challenges related to treatment specificity and drug development. Looking to the future, this review discusses the prospects of ferroptosis-based therapies in CRC, emphasizing the importance of further research to elucidate the interaction between ferroptosis and drug resistance. It proposes future directions for more effective treatment strategies, including the development of new therapeutic approaches, combination therapies, and integration with emerging fields such as precision medicine. In conclusion, harnessing ferroptosis represents a promising avenue for overcoming drug resistance in CRC. Continued research efforts in this field are crucial for optimizing therapeutic outcomes and providing hope for CRC patients.

## 1. Introduction

Drug resistance presents a pervasive challenge in colorectal cancer (CRC) treatment [1,2]. Despite significant advancements in chemotherapy and targeted therapies, the emergence of resistance to commonly used drugs remains a formidable obstacle [3]. Chemotherapeutic agents such as 5-fluorouracil (5-FU) and oxaliplatin, as well as targeted therapies including anti-epidermal growth factor receptor (EGFR) antibodies and anti-vascular endothelial growth factor (VEGF) agents, are extensively employed in CRC treatment. However, prolonged drug exposure can lead to the development of resistance, compromising patient survival and treatment efficacy. The mechanisms underlying drug resistance in CRC are complex and multifactorial, encompassing genetic alterations, activation of survival signaling pathways, and disruptions in drug metabolism and efflux [4,5]. Addressing drug resistance in CRC is crucial for improving patient outcomes and devising effective therapeutic strategies.

The emergence of drug resistance poses a substantial challenge in the management of metastatic colorectal cancer (mCRC). Despite advances in therapeutic approaches, the five-year survival rate for mCRC remains relatively low, primarily due to the development of drug resistance [6]. Approximately half of mCRC patients exhibit resistance to standard chemotherapies like 5-FU [7]. While monoclonal antibodies targeting EGFR, such as cetuximab and panitumumab, have demonstrated efficacy in a subset of patients, the clinical benefit is often limited by the eventual onset of drug resistance. For instance, responders to anti-EGFR monoclonal antibodies experience a relatively short duration of clinical benefit, typically lasting only 8–10 months, with approximately 80% of initial responders eventually developing drug resistance [8,9]. This resistance phenomenon not only leads to treatment failures but also results in tumor relapse, profoundly affecting patient outcomes. Venook et al. conducted a randomized clinical trial that highlighted the adverse impact of drug resistance on overall survival and the quality of life in patients with KRAS wild-type advanced or metastatic CRC [10]. In essence, the consequences of drug resistance are profound, undermining the efficacy of therapeutic interventions and significantly compromising patient prognosis and well-being. By elucidating the underlying mechanisms of drug resistance and developing novel therapeutic strategies, we can mitigate its adverse impact on CRC patients, offering more effective treatment modalities and achieving superior clinical outcomes.

Ferroptosis, as a promising therapeutic approach, operates on distinct principles [11]. It involves a specific form of cell death regulated by iron ions. Ferroptosis differs from conventional treatments by inducing cancer cell death through lipid peroxidation and oxidative stress accumulation [12]. It relies on the intricate balance and regulation of iron metabolism within cells [13]. This unique mechanism provides a potential avenue for targeted intervention against drug-resistant CRC. Understanding and harnessing the principles of ferroptosis can lead to the development of novel therapeutic strategies to overcome drug resistance and improve treatment outcomes in CRC. Ferroptosis holds promise for alternative and effective CRC management.

Elucidating the connection between ferroptosis and drug resistance in CRC is crucial. Exploring the role of ferroptosis in the development and maintenance of drug resistance can provide valuable insights. Ferroptosis, as a novel therapeutic approach, has the potential to bypass drug resistance mechanisms and effectively target drug-resistant tumor cells [2]. The interplay between ferroptosis and drug resistance involves intricate molecular pathways and mechanisms [14]. Understanding the intersection of ferroptosis with drug resistance pathways can inform the development of strategies to overcome resistance and improve treatment outcomes in CRC. By harnessing the unique characteristics of ferroptosis, novel therapeutic interventions can be designed to selectively induce cell death in drug-resistant CRC cells, circumventing their resistance mechanisms and offering new avenues for more effective treatments.

## 2. Mechanisms of Drug Resistance in CRC

### 2.1. Gene Mutations

In CRC, gene mutations have a substantial impact on the development of drug resistance. Several prevalent gene mutations have been identified that contribute to resistance against key therapeutic targets. For example, mutations in the KRAS gene are associated with resistance to anti-EGFR antibodies like cetuximab and panitumumab in CRC patients [15,16,17]. Moreover, alterations in drug-metabolizing enzymes such as UDP-glucuronosyltransferases (UGTs) and cytochrome P450 (CYP) enzymes can influence the metabolism and efficacy of chemotherapeutic agents like irinotecan [18,19,20,21]. The abnormal expression or function of drug transport proteins, such as ATP-binding cassette (ABC) transporters, can also lead to reduced intracellular drug accumulation and confer resistance to various anticancer drugs [22,23]. Understanding the impact of gene mutations on drug resistance mechanisms can facilitate the development of targeted therapies and personalized treatment approaches for CRC patients.

### 2.2. Altered Cell Signaling Pathways

Altered cell signaling pathways play a critical role in the development of drug resistance in CRC [24]. The activation of proliferative and survival signaling pathways within cancer cells, such as the PI3K/AKT/mTOR and MAPK/ERK pathways, can promote cell growth and impede apoptosis, contributing to drug resistance [25,26,27]. For instance, the upregulation of the PI3K/AKT/mTOR pathway has been linked to resistance against targeted therapies like cetuximab in CRC [28,29]. Additionally, changes in apoptotic escape mechanisms, such as the increased expression of anti-apoptotic proteins (e.g., Bcl-2) or decreased expression of pro-apoptotic proteins (e.g., Bax), can impede cancer cell death and confer resistance to chemotherapy [30,31,32,33]. Moreover, abnormalities in DNA repair pathways like mismatch repair (MMR) and homologous recombination (HR) can lead to genomic instability and resistance to DNA-damaging agents [34,35,36].

The development of drug resistance in CRC is intricately linked to calcium signaling pathways [37]. Studies have revealed the significant role of the TRPC5 channel, whose overexpression has been associated with CRC cells’ resistance to 5-FU treatment [38,39]. This suggests TRPC5’s potential as a marker for 5-FU resistance in CRC, influencing the expression of downstream drug resistance proteins like ATP-binding cassette subfamily B1 (ABCB1) and glucose transporter 1 (GLUT1) [40,41]. Sorcin, a mitochondrial isoform, plays a role in anti-apoptosis and MDR across various cancer types by binding to calcium [42,43]. In CRC cells, both the 18-kDa and 22-kDa Sorcin isoforms have been found to modulate drug resistance by preventing endoplasmic reticulum (ER) stress [44]. The 22-kDa Sorcin isoform, in particular, is upregulated under high calcium concentrations in the ER, contributing to resistance against 5-FU, oxaliplatin, and irinotecan [44]. Furthermore, calcium-sensing receptor (CaSR) activation has been linked to enhanced sensitivity of CRC cells to chemotherapeutic agents like mitomycin C and fluorouracil. The level of CaSR expression can impact the differentiation of colon cancer cells, influencing their sensitivity to chemotherapy [45].

Understanding these changes can guide the development of targeted therapies that counteract these signaling alterations, ultimately improving treatment outcomes in CRC patients.

### 2.3. Tumor Microenvironment

The tumor microenvironment (TME) plays a crucial role in the development of drug resistance in CRC [46]. The TME consists of various stromal cell types, such as fibroblasts, immune cells, and mesenchymal stem cells, which interact with tumor cells and contribute to therapeutic resistance [46,47]. These stromal cells within the TME are genetically stable, making them attractive therapeutic targets with reduced risk of resistance and tumor recurrence [46]. One mechanism of drug resistance in CRC is the activation of autophagy, a cellular process that promotes cell survival under stress conditions [48]. Autophagy has been highlighted as a potential therapeutic target to overcome chemotherapy resistance [48]. Additionally, exosomal noncoding RNAs present in the TME have been implicated in tumor drug resistance [49]. The TME also influences drug resistance through its impact on tumor cell proliferation, survival, and signaling pathways [47]. Cancer-associated fibroblasts within the TME provide a supportive microenvironment for cancer cells, leading to therapeutic resistance [47]. Furthermore, the TME can elicit innate resistance to targeted therapies [50]. Epithelial–mesenchymal transitions (EMTs), a process associated with increased invasiveness and drug resistance, can be induced by changes in the TME. The TME can also modulate the immune response, which can impact therapeutic outcomes [51]. Additionally, interactions between cancer cells and their surrounding stromal cells, such as cancer-associated fibroblasts (CAFs) or tumor-associated macrophages (TAMs), create a supportive niche that enhances tumor cell survival and contributes to therapy resistance [52,53]. Understanding these influences can guide the development of strategies targeting the tumor microenvironment to overcome drug resistance and improve treatment outcomes in CRC patients.

Drug resistance stands out as a pivotal factor contributing to treatment failure in CRC. It is crucial to underscore that drug resistance significantly limits the effectiveness of therapies, resulting in tumor recurrence and progression during treatment. Despite advancements in therapeutic approaches, the emergence of drug-resistant cancer cells impedes the ability of drugs to effectively eradicate tumors. This phenomenon not only compromises the initial response to treatment but also poses long-term management challenges. The presence of drug resistance underscores the need for a comprehensive understanding of its underlying mechanisms and the development of innovative strategies to overcome this obstacle. By addressing drug resistance as the primary factor contributing to treatment failure in CRC, we can strive to improve patient outcomes and advance the success of therapeutic interventions.

## 3. Mechanisms of Ferroptosis

**Figure 1:**
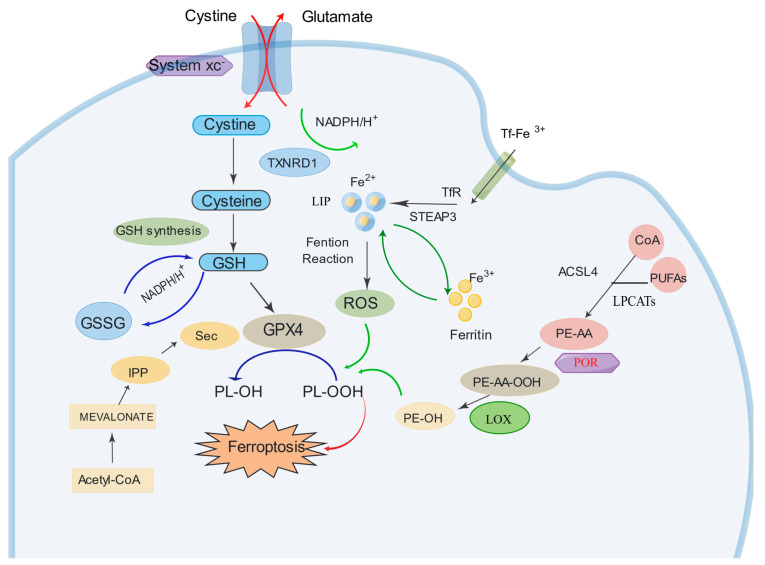
Classic signaling pathways of ferroptosis. Illustration depicting the fundamental signaling pathways associated with ferroptosis. The figure outlines the canonical GPX4-regulated pathway, focusing on the role of GPX4 in lipid peroxide reduction, the iron metabolism pathway elucidating the iron-dependent aspects of ferroptosis, and the lipid metabolism pathway highlighting lipid peroxidation and its contribution to cell membrane rupture. These interconnected pathways underscore the multifaceted nature of ferroptotic cell death. The figures were created using the Figdraw software (https://www.figdraw.com, accessed on 13 August 2023).

Ferroptosis is a unique form of regulated cell death characterized by the iron-dependent accumulation of lipid peroxides, leading to oxidative damage and cell membrane rupture [54]. It is distinct from other cell death modalities, such as apoptosis and necrosis, and is driven by dysregulation in pathways involved in iron metabolism, lipid peroxidation, and antioxidant defense. Key players in ferroptosis include glutathione peroxidase 4 (GPX4), which protects cells from lipid peroxidation, and system Xc-, a cystine/glutamate antiporter involved in maintaining intracellular redox balance [55]. Inclusion of a diagram depicting the iron death pathway can be found in Figure 1. Understanding the mechanisms of ferroptosis can provide insights into its role in various diseases and pave the way for the development of novel therapeutic strategies targeting this form of cell death.

### 3.1. Canonical GPX4-Regulated Pathway

The canonical GPX4-regulated pathway plays a pivotal role in the induction of ferroptosis, as revealed by the study conducted by Yang et al. [56]. Their findings demonstrated that the direct or indirect inhibition of GPX4 through glutathione (GSH) depletion is essential for triggering ferroptosis [56]. GPX4, functioning as a glutathione peroxidase, is responsible for catalyzing the conversion of phospholipid hydroperoxides (PLOOHs) into phospholipid alcohols, a critical process in maintaining cell membrane integrity. The availability of intracellular GSH, which is regulated by the cystine/glutamate antiporter (xCT) system and glutamate–cysteine ligase, influences GPX4 activity and determines the susceptibility to ferroptosis [57]. Inactivation of GPX4 leads to the accumulation of PLOOHs, resulting in cellular membrane damage and subsequent ferroptotic cell death [58]. Acetyl-CoA and the MEVALONATE pathway also play crucial roles in cells and influence their sensitivity to ferroptosis [59,60]. Isopentenyl pyrophosphate (IPP) serves as a pivotal intermediate in this process, participating in the biosynthesis of cell membranes, which is directly related to the occurrence of ferroptosis [60]. Additionally, selenium (Sec) functions as a cofactor for GPX4, a key protein in the ferroptosis pathway [61]. GPX4 maintains cell membrane integrity by reducing lipid peroxides, thus inhibiting the onset of ferroptosis. Targeting the canonical GPX4-regulated pathway shows great promise as a therapeutic strategy for selectively inducing ferroptosis in cancer cells. Further research efforts are warranted to deepen our understanding of this pathway and develop innovative interventions to exploit the therapeutic potential of ferroptosis in cancer treatment.

### 3.2. Iron Metabolism Pathway

Iron, particularly in its Fe^2+^ form, is a central player in driving ferroptosis through the generation of reactive oxygen species (ROS) via the Fenton reaction [62]. This reaction involves the interaction of Fe^2+^ with hydrogen peroxide (H_2_O_2_), leading to the production of highly reactive hydroxyl radicals (•OH). These hydroxyl radicals, in turn, initiate lipid peroxidation, a hallmark of ferroptosis, by attacking polyunsaturated fatty acids (PUFAs) in cell membranes [62]. Transferrin receptors (TFR) and ferritin, key components of the iron metabolism pathway, play significant roles in regulating intracellular iron levels. TFR facilitates the uptake of iron by binding to transferrin, which transports Fe^3+^ into the cell via receptor-mediated endocytosis [63]. Ferritin, on the other hand, acts as an iron storage protein, sequestering excess intracellular iron and preventing it from participating in the Fenton reaction [64]. This delicate balance between iron uptake and storage is crucial for determining a cell’s susceptibility to ferroptosis.

Furthermore, the role of iron transporters, such as STEAP3 (six-transmembrane epithelial antigen of prostate 3), in facilitating iron uptake and releasing Fe^2+^ into the labile iron pool (LIP) cannot be overstated [65,66]. The LIP is a critical reservoir of Fe^2+^ that fuels the Fenton reaction and subsequently drives lipid peroxidation, a key event in ferroptosis [67]. Understanding the complex interactions between iron metabolism, ROS generation, and lipid peroxidation is critical for developing targeted interventions that exploit the vulnerabilities of cancer cells and enhance the effectiveness of ferroptosis-based therapies.

### 3.3. Lipid Metabolism Pathway

Ferroptosis is primarily driven by the peroxidation of membrane phospholipids, resulting in the formation of PLOOHs, which further decompose into 4-hydroxynonenal or malondialdehyde [68,69]. The accumulation of lipid peroxidation products induces membrane instability and permeabilization, ultimately leading to cell death [70].

In nonenzymatic lipid peroxidation, the conversion of polyunsaturated fatty acids (PUFAs) into acyl-CoA is facilitated by acyl-CoA synthetase long-chain family member 4 (ACSL4), which ligates PUFAs with coenzyme A (CoA) [59]. These acyl-CoA molecules can then be re-esterified into phospholipids by lysophosphatidylcholine acyltransferases (LPCATs), forming phospholipids. Therefore, the regulation of ACSL4 and LPCATs plays a critical role in determining the sensitivity to ferroptosis [69]. Enzymatic lipid peroxidation involves the activity of lipoxygenases (LOX) and cytochrome P450 oxidoreductase (POR) [71]. LOX enzymes, which contain nonheme iron, directly catalyze the deoxygenation of free and esterified PUFAs, resulting in the production of PLOOHs. Studies have demonstrated that overexpression of specific LOX isoforms increases cellular susceptibility to ferroptosis, while LOX inhibitors act as effective antioxidants, protecting cells from lipid peroxidation [72]. In 2020, Zou et al. identified POR as a crucial mediator of ferroptotic cell death in cancer cells through genome-wide CRISPR-Cas9 screens [73]. Previous research has suggested that P450 enzymes can accept electrons from POR and catalyze the peroxidation of PUFAs [74,75]. The pro-ferroptotic role of POR has been further supported by genetic depletion experiments across different cell types and lineages.

Understanding the intricate dynamics of the lipid metabolism pathway is essential for unraveling the mechanisms underlying ferroptosis. Further research in this field will enhance our knowledge of the regulatory mechanisms of lipid peroxidation and identify potential targets for therapeutic interventions aimed at promoting or inhibiting ferroptosis. These insights offer new avenues for the development of innovative strategies for cancer treatment.

## 4. Role of Ferroptosis in CRC and Its Impact on Drug Resistance

**Figure 2:**
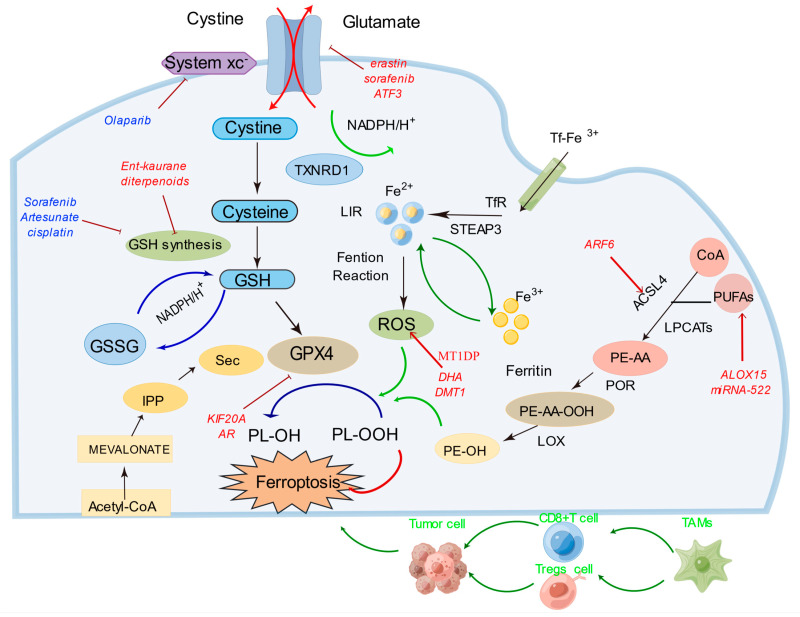
Mechanisms of drug resistance reversal through ferroptosis targeting. This figure illustrates diverse molecular interactions and sites of action for various agents aimed at reversing drug resistance through ferroptosis modulation. In the diagram, red text denotes sites of action for agents targeting ferroptosis reversal in chemotherapy, blue text represents sites of action for agents targeting ferroptosis reversal specifically, and green text indicates sites of action for agents targeting ferroptosis reversal in immunotherapy. The integrated approach to targeting these pathways highlights their potential synergistic effects in overcoming drug resistance. The figures were created using the Figdraw software (https://www.figdraw.com, accessed on 13 August 2023).

Ferroptosis has garnered significant attention in CRC research, offering novel insights and strategies for CRC treatment and prognosis. The relationships between certain genes and ferroptosis in CRC are illustrated in Figure 2. Elevated expression levels of several ferroptosis-related factors have been observed in CRC tissues, including TIGAR, AADAC, and a series of ferroptosis-related genes (FRGs) [76,77]. The dysregulated expression of these factors is closely associated with clinical outcomes in CRC patients, providing crucial prognostic and therapeutic predictive indicators [78,79]. Furthermore, researchers have discovered that by modulating the ferroptosis pathway, it is possible to enhance the sensitivity of CRC cells to specific drugs such as erastin and RSL3 [80,81]. These drugs induce ferroptosis in CRC cells, presenting a novel approach to CRC therapy. Some studies have identified gene signatures related to ferroptosis, including ACACA, GSS, and NFS1, among others, which have been validated as independent prognostic factors for CRC, outperforming the traditional TNM staging system in survival prediction [79]. The aberrant expression of these genes is closely correlated with CRC development and treatment response, offering diversified therapeutic options for patients.

On a specific gene and protein level, certain studies emphasize the roles of particular factors in CRC, such as KRAS mutations, Nodal, MT1G, SFRS9, and p53 [82,83,84,85,86]. The dysregulated expression of these factors is intricately linked to CRC progression and drug resistance, rendering them potential targets for future therapeutic interventions. Additionally, researchers have explored novel approaches to modulate the ferroptosis pathway, including siRNA nanoparticles, GCH1 inhibitors, and the CUL9-HNRNPC-MATE1 negative feedback loop, with the potential to enhance the sensitivity of CRC cells to ferroptosis inducers and overcome drug resistance [87,88,89]. Furthermore, miRNAs and circular RNAs (circRNAs) play pivotal roles in CRC by influencing the development of the disease through the regulation of ferroptosis-related pathways [90,91,92]. Collectively, these studies reveal the diversity and complexity of ferroptosis in CRC, providing valuable clues and directions for future research and treatment. In conclusion, research on ferroptosis in CRC has made significant progress, spanning multiple levels, from molecular mechanisms to potential therapeutic strategies, all contributing to a deeper understanding of CRC’s development and treatment. These studies offer substantial support for future personalized treatments and the overcoming of treatment resistance.

## 5. Potential Therapeutic Approaches Targeting Ferroptosis

Ferroptosis stands as a promising frontier in CRC therapy, offering a new perspective on overcoming the hurdles of treatment resistance. As we delve into the intricate mechanisms underlying these approaches, Figure 2 serves as a comprehensive visual guide, shedding light on the potential synergies between targeted drug interventions, immunotherapy, and the induction of ferroptosis. In the following sections, we will elaborate on the interplay between traditional chemotherapy drug resistance and ferroptosis, the intriguing relationship between targeted drug resistance and the ferroptosis process, and how the realm of immunotherapy intertwines with ferroptosis to shape the future of cancer treatment paradigms.

### 5.1. Targeted Drug Therapy for Ferroptosis

Chemotherapy has remained an essential approach in cancer treatment, but drug resistance remains a significant factor contributing to poor patient prognosis. Ferroptosis, with its molecular mechanisms, plays a crucial role in reducing chemotherapeutic drug resistance. Pathways involved in lipid metabolism, iron metabolism, and the classical GPX4 pathway are implicated in drug resistance in CRC and other malignancies. In lipid metabolism, ACSL4 participates in the lipid oxidation pathway by converting the AA and AdA in PUFAs into coenzyme derivatives, leading to the production of oxidized lipid molecules [93,94]. Another enzyme, LOX, mediates ferroptosis non-enzymatically by directly oxidizing PUFAs. ALOX15, a key player in gastric cancer, inhibits ferroptosis [95,96]. Decreasing miRNA-522 and increasing ALOX15 has emerged as a novel treatment strategy to reverse drug resistance, particularly resistance to cisplatin/paclitaxel [95].

Iron metabolic pathways are also involved in reversing drug resistance. Dihydroartemisinin (DHA), a safe and promising therapeutic agent, selectively induces ferroptosis in cancer cells. DHA intensifies the cytotoxicity of cisplatin by impairing mitochondrial homeostasis, increasing mitochondrial-derived ROS, and promoting ferroptosis through the accumulation of free iron and lipid peroxidation [97,98]. Blocking lysosomal iron translocation through the inhibition of DMT1 in CSC leads to iron accumulation in lysosomes, ROS production, and ferroptosis-mediated cell death [99,100]. Furthermore, indirectly triggering ferroptosis by blocking GSH synthesis or inhibiting system Xc- enhances the reversal of drug resistance. Ent-kaurane diterpenoids overcome cisplatin resistance by targeting peroxiredoxin I/II and consuming GSH to induce ferroptosis [101]. In head and neck cancer, inhibiting system Xc- and the Nrf2/Keap1/system Xc- signaling pathway can overcome cisplatin resistance [102,103].

In parallel, a series of studies has underscored the potential of targeted drug therapy for ferroptosis in CRC. For instance, adipose-derived exosomes upregulate the microsomal triglyceride transfer protein (MTTP), reducing ferroptosis susceptibility and contributing to oxaliplatin resistance [104]. Aspirin enhances the sensitivity of CRC cells with oncogenic PIK3CA activation to ferroptosis induction by inhibiting the AKT/mTOR pathway, suppressing SREBP-1 expression, and reducing SCD1-mediated lipogenesis [105]. Loss of the metabolic enzyme NFS1 in CRC cells heightens sensitivity to oxaliplatin and induces PANoptosis [106]. KIF20A, associated with oxaliplatin resistance, becomes a potential target for sensitizing CRC cells to this drug [107]. Moreover, Lipocalin 2 overexpression in colon cancer cells has conferred resistance to 5-fluorouracil through ferroptosis inhibition. FAM98A overexpression in CRC tissues has been linked to drug resistance, with metformin demonstrating potential in reversing FAM98A-mediated 5-FU resistance [108]. The cyclin-dependent kinase 1 (CDK1) has been identified as a key contributor to oxaliplatin resistance in CRC, highlighting the prospect of CDK1 inhibitors for treating oxaliplatin-resistant CRC patients [109]. Additionally, PYCR1, an oncogenic gene, has been associated with reducing the sensitivity of CRC cells to 5-fluorouracil and promoting tumor growth [110].

In summary, targeted drug therapy for ferroptosis holds immense potential in overcoming chemotherapy resistance in CRC. By addressing key molecular mechanisms and therapeutic targets, researchers aim to sensitize CRC cells to ferroptosis-inducing agents, ultimately improving treatment outcomes for CRC patients. These findings collectively provide a multifaceted approach to combatting drug resistance and enhancing the efficacy of CRC therapies.

### 5.2. Targeted Therapy for Ferroptosis

In contrast to chemotherapy, targeted therapy has emerged as an effective treatment with fewer side effects on normal cells. For example, the presence of RAS mutations in about half of metastatic CRC patients greatly limits the efficacy of cetuximab. β-elemene, a natural product derived from turmeric, combined with cetuximab, exhibits high cytotoxicity toward metastatic CRC cells with KRAS mutations [111]. This combination therapy induces ferroptosis and inhibits the epithelial–mesenchymal transition. Olaparib, a well-known poly (ADP-ribose) polymerase inhibitor, promotes ferroptosis by inhibiting SLC7A11-mediated GSH synthesis [112]. Combined with FINs, it sensitizes BRCA-activated ovarian cancer cells and xenografts [112]. In TNBC cells resistant to gefitinib, inhibiting and inducing ferroptosis enhances sensitivity to gefitinib [113]. Sorafenib, the first approved systemic medicine for advanced hepatocellular carcinoma, faces acquired resistance. Similarly, like erastin, cisplatin resistance can be overcome by depleting GSH accompanied by GPx inactivation. Combining erastin with cisplatin demonstrates enhanced antitumor activity compared to cisplatin alone, as their mechanisms of action differ [95,114].

Metallothionein (MT) is a multifunctional protein that plays a pivotal role in various aspects of ferroptosis regulation, drug resistance, ROS elimination, and Fe2+ metabolism. In the context of CRC, MT1G has emerged as a significant factor influencing ferroptosis susceptibility, contributing to drug resistance and disease progression. MT1G downregulation in clear cell renal cell carcinoma is associated with advanced stages and higher malignancy, potentially due to its negative regulatory role in ferroptosis and its influence on GSH metabolism [115]. Furthermore, in hepatocellular carcinoma, MT-1G is identified as a key player in the regulation of ferroptosis and is associated with sorafenib resistance. This highlights the importance of MTs in HCC treatment and potential strategies for overcoming resistance [116]. Additionally, in leukemia, MTs, particularly the MT2A and MT1M isoforms, are implicated in the modulation of ferroptosis induced by DHA [117]. Their role in regulating iron metabolism and cellular antioxidant responses underscores their impact on leukemic cell susceptibility to ferroptotic cell death. Moreover, metallothionein 1D pseudogene (MT1DP) is shown to be a crucial regulator of ferroptosis in non-small cell lung cancer (NSCLC), sensitizing cancer cells to erastin-induced ferroptosis by downregulating NRF2 and enhancing oxidative stress. This novel therapeutic strategy holds promise for the treatment of lung cancer [118]. Furthermore, MTs’ interaction with ferritin, a protein involved in iron storage, is suggested to have a potential impact on iron release under oxidative conditions. In the context of Parkinson’s disease (PD), where iron accumulation is linked to ferroptosis and disease pathogenesis, understanding the interplay between MTs and iron metabolism may offer insights into potential neuroprotective strategies [119].

Targeted therapy for ferroptosis offers innovative strategies for sensitizing cancer cells to ferroptosis-inducing agents, with a focus on KRAS-mutant CRC. Combining cetuximab with RSL3 enhances ferroptosis in KRAS-mutant CRC cells, providing a strategy to overcome drug resistance in this specific subgroup [120]. Additionally, apatinib has been identified as a ferroptosis promoter in CRC cells by targeting the ELOVL6/ACSL4 pathway, suggesting its potential as a valuable addition to CRC treatment strategies [121]. A novel ferroptosis inducer, talaroconvolutin A (TalaA), has demonstrated remarkable efficacy in suppressing CRC growth in mouse models, positioning it as a potent candidate for CRC therapy via ferroptosis induction [122]. Lastly, co-treatment with 3-Bromopyruvate (3-BP) and cetuximab has emerged as a promising strategy to overcome cetuximab resistance in CRC by inducing ferroptosis synergistically [123].

In summary, targeted therapy for ferroptosis offers an innovative and effective approach to combat drug resistance and enhance treatment outcomes in various cancer types, including CRC. These strategies hold promise for improving the prognosis and quality of life for cancer patients while minimizing the impact on healthy cells.

### 5.3. Immunotherapy Targeting Ferroptosis

Immunotherapy has emerged as a promising approach for cancer treatment, and recent research has shown that it can also regulate ferroptosis. Wang et al. found that CD8+ T cells activated by immunotherapy enhance ferroptosis-specific lipid peroxidation in tumor cells, leading to increased tumor cell death and improved anti-tumor efficacy [124]. This suggests that targeting ferroptosis could enhance the effectiveness of immunotherapy in cancer treatment. The mechanistic link between ferroptosis and cancer has been further explored in recent studies. One paper reviewed the regulatory mechanisms of mTORC1 and ferroptosis and proposed co-targeting mTOR and ferroptosis as a potential strategy for cancer treatment [125]. Another investigated ferroptosis as an autophagic cell death process and highlighted its relevance in cancer and cancer treatment [126]. One study reviewed the development of agents targeting molecules involved in ferroptosis, emphasizing the potential of ferroptosis as a therapeutic strategy for cancer [127]. A further study discussed the epigenetic regulators and metabolic changes associated with ferroptosis in cancer progression, suggesting that targeting ferroptosis-associated metabolism could improve the efficacy of cancer immunotherapy [128]. Others have demonstrated that immunotherapy sensitizes tumors to radiotherapy by promoting tumor cell ferroptosis, further supporting the potential synergy between immunotherapy and ferroptosis in cancer treatment [129].

The role of ferroptosis in cancer immunotherapy has also been recognized. One study highlighted ferroptosis as an effector pathway for cancer immunotherapy [130]. Another investigated the interaction between ferroptosis and immunotherapy in cancer cells and found that ferroptosis enhances the anti-tumor efficacy of immunotherapy through increased ferroptosis-specific lipid peroxidation and reduced cystine uptake induced by immunotherapy-activated CD8+ T cells [131]. Combining radiotherapy with PARP inhibitors, such as Niraparib, activates the cGAS signaling pathway and enhances ferroptosis, promoting an anti-tumor immune response [132]. Apolipoprotein L3 (APOL3) has been identified as a key modulator, positively affecting sensitivity to ferroptosis and improving CD8+ T cell-mediated anti-tumor responses in CRC [133]. Co-treatment with PR-619 and anti-PD1 inhibits CRC growth, induces ferroptosis, and enhances CD8+ T cell-mediated immunity [134]. Moreover, inhibiting CYP1B1, which contributes to ferroptosis resistance, enhances the sensitivity of CRC cells to anti-PD-1 antibody therapy [135]. These immunotherapy approaches can synergize with ferroptosis induction by enhancing the immune system’s recognition and clearance of cancer cells undergoing ferroptotic cell death.

However, it is important to consider the limitations of immunotherapy targeting ferroptosis. Some tumors employ immune evasion mechanisms, such as downregulation of antigen presentation or upregulation of immunosuppressive factors, which can hinder the immune response and limit the effectiveness of immunotherapy. Additionally, tumor heterogeneity and individual variations in immune responses can impact the outcomes of immunotherapeutic interventions. Further research is needed to better understand these limitations and develop strategies to overcome them.

## 6. The Advantages and Limitations of Therapeutic Approaches Targeting Ferroptosis

Therapeutic strategies targeting ferroptosis offer several advantages compared to traditional treatment methods. One significant advantage is their high selectivity towards cancer cells, which can minimize damage to healthy tissues and reduce treatment-related side effects [136]. Additionally, ferroptosis induction may hold potential for overcoming drug resistance mechanisms, as it represents a distinct form of cell death that can bypass common resistance pathways. Moreover, targeting iron metabolism and lipid peroxidation, the core processes of ferroptosis, provides a unique opportunity to exploit vulnerabilities specific to cancer cells.

However, these therapeutic approaches targeting ferroptosis may also face certain challenges. One challenge is achieving treatment specificity and ensuring that the intervention selectively targets cancer cells without affecting normal cells. Another challenge lies in the development of effective and safe drugs that can modulate ferroptosis. The complex interplay of iron metabolism and lipid peroxidation pathways adds to the intricacy of drug development and requires thorough understanding to maximize therapeutic efficacy [136]. Furthermore, the potential irreversibility of ferroptotic cell death raises concerns regarding tissue damage and long-term effects.

## 7. Paving the Way for Future Developments in Therapeutic Approaches Targeting Ferroptosis

To further improve treatment outcomes and overcome the existing limitations, future research should focus on multiple fronts. Firstly, there is a need for continued exploration and development of novel therapeutic strategies that can specifically target ferroptosis. This includes the identification of new ferroptosis inducers and the refinement of existing ones to enhance their efficacy and safety profiles. Secondly, combining ferroptosis-based approaches with other treatment modalities, such as immunotherapy, targeted therapy, or precision medicine, holds great potential in optimizing treatment outcomes [137]. These synergistic combinations can exploit different pathways and vulnerabilities, leading to enhanced therapeutic efficacy.

Additionally, future developments should encompass the integration of ferroptosis-targeting strategies with emerging fields such as personalized medicine and precision oncology. Understanding the interplay between ferroptosis and specific molecular subtypes of cancer, as well as identifying predictive biomarkers, can enable the selection of appropriate patients and tailored treatment regimens for maximum effectiveness. The exploration of non-invasive imaging techniques to assess ferroptosis status in tumors and the development of strategies to modulate ferroptosis in a controlled manner are also promising directions for future research.

In conclusion, therapeutic approaches targeting ferroptosis offer distinct advantages, including selectivity, low side effects, and potential for overcoming drug resistance. However, challenges related to treatment specificity, drug development, and potential irreversibility exist. Future directions should focus on refining therapeutic strategies, exploring combination therapies, and integrating ferroptosis-based approaches with personalized medicine to optimize treatment outcomes and overcome current limitations.

## 8. Conclusions and Perspectives

The potential of targeting ferroptosis as a therapeutic strategy to overcome drug resistance in CRC is of significant importance. Ferroptosis, as a distinct form of cell death, offers unique advantages in selectively eliminating cancer cells and bypassing common resistance mechanisms. This detailed exploration of ferroptosis’s role in CRC, its mechanisms, and its interplay with drug resistance mechanisms underscores the necessity for further research in this field. Promising advances have been made in identifying ferroptosis inducers, elucidating key regulators, and exploring combination therapies. However, there are challenges to address, including treatment specificity and the development of safe and effective drugs. Future directions should focus on refining therapeutic strategies, such as the development of novel ferroptosis inducers and the integration of ferroptosis-based approaches with other treatment modalities. Moreover, the incorporation of ferroptosis research into the realm of personalized medicine holds great potential. By understanding the complex interplay between ferroptosis and resistance mechanisms, identifying predictive biomarkers, and refining treatment selection, we can optimize therapeutic outcomes and provide hope for CRC patients. Continued research efforts in this field will pave the way for more effective treatment strategies and better outcomes in CRC management.

## Abbreviations

PUFAs	polyunsaturated fatty acids	CRC	colorectal cancer
CoA	coenzyme A	5-FU	5-fluorouracil
LPCATs	Lys phosphatidylcholine acyltransferases	EGFR	epidermal growth factor receptor
LOX	lipoxygenases	VEGF	vascular endothelial growth factor
POR	P450 oxidoreductase	UGTs	UDP-glucuronosyltransferases
FRGs	ferroptosis-related genes	CYP	cytochrome P450
DHA	Dihydroartemisinin	ABC	ATP-binding cassette
MTTP	microsomal triglyceride transfer protein	MMR	mismatch repair
CDK1	cyclin-dependent kinase 1	HR	homologous recombination
MT	Metallothionein	MHC	major histocompatibility complex
MT1DP	metallothionein 1D pseudogene	CAFs	cancer-associated fibroblasts
TalaA	talaroconvolutin A	TAMs	tumor-associated macrophages
TME	tumor microenvironment	GPX4	glutathione peroxidase 4
TAMs	tumor-associated macrophages	GSH	glutathione
APOL3	Apolipoprotein L3	PLOOHs	phospholipid hydroperoxides
ABCB1	ATP-binding cassette subfamily B1	xCT	cystine/glutamate antiporter
CaSR	calcium-sensing receptor	ROS	reactive oxygen species
TME	tumor microenvironment	GLUT1	glucose transporter 1
EMT	Epithelial–mesenchymal transition	ACSL4	acyl-CoA synthetase long-chain family member 4

## References

[1] Luo M., Yang X., Chen H.-N., Nice E.C., Huang C. (2021). Drug resistance in colorectal cancer: An epigenetic overview. Biochim. Biophys. Acta Rev. Cancer.

[2] Friedmann Angeli J.P., Krysko D.V., Conrad M. (2019). Ferroptosis at the crossroads of cancer-acquired drug resistance and immune evasion. Nat. Rev. Cancer.

[3] Hutchinson L. (2012). Colorectal cancer: A step closer to combating acquired resistance in CRC. Nat. Rev. Gastroenterol. Hepatol..

[4] Wang Q., Shen X., Chen G., Du J. (2022). Drug Resistance in Colorectal Cancer: From Mechanism to Clinic. Cancers.

[5] Martini G., Ciardiello D., Vitiello P.P., Napolitano S., Cardone C., Cuomo A., Troiani T., Ciardiello F., Martinelli E. (2020). Resistance to anti-epidermal growth factor receptor in metastatic colorectal cancer: What does still need to be addressed?. Cancer Treat. Rev..

[6] Van Cutsem E., Cervantes A., Adam R., Sobrero A., Van Krieken J.H., Aderka D., Aranda Aguilar E., Bardelli A., Benson A., Bodoky G. (2016). ESMO consensus guidelines for the management of patients with metastatic colorectal cancer. Ann. Oncol. Off. J. Eur. Soc. Med. Oncol..

[7] Yaffee P., Osipov A., Tan C., Tuli R., Hendifar A. (2015). Review of systemic therapies for locally advanced and metastatic rectal cancer. J. Gastrointest. Oncol..

[8] Tabernero J., Van Cutsem E., Díaz-Rubio E., Cervantes A., Humblet Y., André T., Van Laethem J.-L., Soulié P., Casado E., Verslype C. (2007). Phase II trial of cetuximab in combination with fluorouracil, leucovorin, and oxaliplatin in the first-line treatment of metastatic colorectal cancer. J. Clin. Oncol. Off. J. Am. Soc. Clin. Oncol..

[9] Borner M., Koeberle D., Von Moos R., Saletti P., Rauch D., Hess V., Trojan A., Helbling D., Pestalozzi B., Caspar C. (2008). Adding cetuximab to capecitabine plus oxaliplatin (XELOX) in first-line treatment of metastatic colorectal cancer: A randomized phase II trial of the Swiss Group for Clinical Cancer Research SAKK. Ann. Oncol. Off. J. Eur. Soc. Med. Oncol..

[10] Venook A.P., Niedzwiecki D., Lenz H.-J., Innocenti F., Fruth B., Meyerhardt J.A., Schrag D., Greene C., O’Neil B.H., Atkins J.N. (2017). Effect of First-Line Chemotherapy Combined With Cetuximab or Bevacizumab on Overall Survival in Patients With KRAS Wild-Type Advanced or Metastatic Colorectal Cancer: A Randomized Clinical Trial. JAMA.

[11] Hassannia B., Vandenabeele P., Vanden Berghe T. (2019). Targeting Ferroptosis to Iron Out Cancer. Cancer Cell.

[12] Jiang X., Stockwell B.R., Conrad M. (2021). Ferroptosis: Mechanisms, biology and role in disease. Nat. Rev. Mol. Cell Biol..

[13] Chen X., Kang R., Kroemer G., Tang D. (2021). Broadening horizons: The role of ferroptosis in cancer. Nat. Rev. Clin. Oncol..

[14] Zhang C., Liu X., Jin S., Chen Y., Guo R. (2022). Ferroptosis in cancer therapy: A novel approach to reversing drug resistance. Mol. Cancer.

[15] Misale S., Yaeger R., Hobor S., Scala E., Janakiraman M., Liska D., Valtorta E., Schiavo R., Buscarino M., Siravegna G. (2012). Emergence of KRAS mutations and acquired resistance to anti-EGFR therapy in colorectal cancer. Nature.

[16] Zhu C., Guan X., Zhang X., Luan X., Song Z., Cheng X., Zhang W., Qin J.-J. (2022). Targeting KRAS mutant cancers: From druggable therapy to drug resistance. Mol. Cancer.

[17] Douillard J.-Y., Oliner K.S., Siena S., Tabernero J., Burkes R., Barugel M., Humblet Y., Bodoky G., Cunningham D., Jassem J. (2013). Panitumumab-FOLFOX4 treatment and RAS mutations in colorectal cancer. N. Engl. J. Med..

[18] Zahreddine H.A., Borden K.L.B. (2015). Molecular Pathways: GLI1-Induced Drug Glucuronidation in Resistant Cancer Cells. Clin. Cancer Res. Off. J. Am. Assoc. Cancer Res..

[19] Mazerska Z., Mróz A., Pawłowska M., Augustin E. (2016). The role of glucuronidation in drug resistance. Pharmacol. Ther..

[20] Zhao M., Ma J., Li M., Zhang Y., Jiang B., Zhao X., Huai C., Shen L., Zhang N., He L. (2021). Cytochrome P450 Enzymes and Drug Metabolism in Humans. Int. J. Mol. Sci..

[21] McFadyen M.C., McLeod H.L., Jackson F.C., Melvin W.T., Doehmer J., Murray G.I. (2001). Cytochrome P450 CYP1B1 protein expression: A novel mechanism of anticancer drug resistance. Biochem. Pharmacol..

[22] Robey R.W., Pluchino K.M., Hall M.D., Fojo A.T., Bates S.E., Gottesman M.M. (2018). Revisiting the role of ABC transporters in multidrug-resistant cancer. Nat. Rev. Cancer.

[23] Hou Y.-Q., Wang Y.-Y., Wang X.-C., Liu Y., Zhang C.-Z., Chen Z.-S., Zhang Z., Wang W., Kong D.-X. (2020). Multifaceted anti-colorectal tumor effect of digoxin on HCT8 and SW620 cells in vitro. Gastroenterol. Rep..

[24] Mashouri L., Yousefi H., Aref A.R., Ahadi A.M., Molaei F., Alahari S.K. (2019). Exosomes: Composition, biogenesis, and mechanisms in cancer metastasis and drug resistance. Mol. Cancer.

[25] Narayanankutty A. (2019). PI3K/ Akt/ mTOR Pathway as a Therapeutic Target for Colorectal Cancer: A Review of Preclinical and Clinical Evidence. Curr. Drug. Targets.

[26] Huang J., Chen L., Wu J., Ai D., Zhang J.-Q., Chen T.-G., Wang L. (2022). Targeting the PI3K/AKT/mTOR Signaling Pathway in the Treatment of Human Diseases: Current Status, Trends, and Solutions. J. Med. Chem..

[27] Pashirzad M., Khorasanian R., Fard M.M., Arjmand M.-H., Langari H., Khazaei M., Soleimanpour S., Rezayi M., Ferns G.A., Hassanian S.M. (2021). The Therapeutic Potential of MAPK/ERK Inhibitors in the Treatment of Colorectal Cancer. Curr. Cancer Drug. Targets.

[28] Yang G., Huang L., Jia H., Aikemu B., Zhang S., Shao Y., Hong H., Yesseyeva G., Wang C., Li S. (2021). NDRG1 enhances the sensitivity of cetuximab by modulating EGFR trafficking in colorectal cancer. Oncogene.

[29] Koustas E., Karamouzis M.V., Mihailidou C., Schizas D., Papavassiliou A.G. (2017). Co-targeting of EGFR and autophagy signaling is an emerging treatment strategy in metastatic colorectal cancer. Cancer Lett..

[30] Guièze R., Liu V.M., Rosebrock D., Jourdain A.A., Hernández-Sánchez M., Martinez Zurita A., Sun J., Ten Hacken E., Baranowski K., Thompson P.A. (2019). Mitochondrial Reprogramming Underlies Resistance to BCL-2 Inhibition in Lymphoid Malignancies. Cancer Cell.

[31] Zhang L., Lu Z., Zhao X. (2021). Targeting Bcl-2 for cancer therapy. Biochim. Biophys. Acta Rev. Cancer.

[32] Nechiporuk T., Kurtz S.E., Nikolova O., Liu T., Jones C.L., D’Alessandro A., Culp-Hill R., d’Almeida A., Joshi S.K., Rosenberg M. (2019). The TP53 Apoptotic Network Is a Primary Mediator of Resistance to BCL2 Inhibition in AML Cells. Cancer Discov..

[33] Hafezi S., Rahmani M. (2021). Targeting BCL-2 in Cancer: Advances, Challenges, and Perspectives. Cancers.

[34] Russo M., Crisafulli G., Sogari A., Reilly N.M., Arena S., Lamba S., Bartolini A., Amodio V., Magrì A., Novara L. (2019). Adaptive mutability of colorectal cancers in response to targeted therapies. Science.

[35] Dong L., Jiang H., Kang Z., Guan M. (2023). Biomarkers for chemotherapy and drug resistance in the mismatch repair pathway. Clin. Chim. Acta.

[36] Ray Chaudhuri A., Callen E., Ding X., Gogola E., Duarte A.A., Lee J.-E., Wong N., Lafarga V., Calvo J.A., Panzarino N.J. (2016). Replication fork stability confers chemoresistance in BRCA-deficient cells. Nature.

[37] Monteith G.R., Prevarskaya N., Roberts-Thomson S.J. (2017). The calcium-cancer signalling nexus. Nat. Rev. Cancer.

[38] Chen Z., Tang C., Zhu Y., Xie M., He D., Pan Q., Zhang P., Hua D., Wang T., Jin L. (2017). TrpC5 regulates differentiation through the Ca2+/Wnt5a signalling pathway in colorectal cancer. Clin. Sci..

[39] Chen Z., Zhu Y., Dong Y., Zhang P., Han X., Jin J., Ma X. (2017). Overexpression of TrpC5 promotes tumor metastasis via the HIF-1α-Twist signaling pathway in colon cancer. Clin. Sci. (Lond.).

[40] Wang T., Ning K., Lu T.-X., Hua D. (2017). Elevated expression of TrpC5 and GLUT1 is associated with chemoresistance in colorectal cancer. Oncol. Rep..

[41] Wang T., Chen Z., Zhu Y., Pan Q., Liu Y., Qi X., Jin L., Jin J., Ma X., Hua D. (2015). Inhibition of transient receptor potential channel 5 reverses 5-Fluorouracil resistance in human colorectal cancer cells. J. Biol. Chem..

[42] Landriscina M., Laudiero G., Maddalena F., Amoroso M.R., Piscazzi A., Cozzolino F., Monti M., Garbi C., Fersini A., Pucci P. (2010). Mitochondrial chaperone Trap1 and the calcium binding protein Sorcin interact and protect cells against apoptosis induced by antiblastic agents. Cancer Res..

[43] Battista T., Fiorillo A., Chiarini V., Genovese I., Ilari A., Colotti G. (2020). Roles of Sorcin in Drug Resistance in Cancer: One Protein, Many Mechanisms, for a Novel Potential Anticancer Drug Target. Cancers.

[44] Maddalena F., Laudiero G., Piscazzi A., Secondo A., Scorziello A., Lombardi V., Matassa D.S., Fersini A., Neri V., Esposito F. (2011). Sorcin induces a drug-resistant phenotype in human colorectal cancer by modulating Ca(2+) homeostasis. Cancer Res..

[45] Chakrabarty S., Radjendirane V., Appelman H., Varani J. (2003). Extracellular calcium and calcium sensing receptor function in human colon carcinomas: Promotion of E-cadherin expression and suppression of beta-catenin/TCF activation. Cancer Res..

[46] Quail D.F., Joyce J.A. (2013). Microenvironmental regulation of tumor progression and metastasis. Nat. Med..

[47] Kadel D., Zhang Y., Sun H.-R., Zhao Y., Dong Q.-Z., Qin L.-X. (2019). Current perspectives of cancer-associated fibroblast in therapeutic resistance: Potential mechanism and future strategy. Cell Biol. Toxicol..

[48] Sui X., Chen R., Wang Z., Huang Z., Kong N., Zhang M., Han W., Lou F., Yang J., Zhang Q. (2013). Autophagy and chemotherapy resistance: A promising therapeutic target for cancer treatment. Cell Death Dis..

[49] Guo C., Liu J., Zhou Q., Song J., Zhang Z., Li Z., Wang G., Yuan W., Sun Z. (2020). Exosomal Noncoding RNAs and Tumor Drug Resistance. Cancer Res..

[50] Straussman R., Morikawa T., Shee K., Barzily-Rokni M., Qian Z.R., Du J., Davis A., Mongare M.M., Gould J., Frederick D.T. (2012). Tumour micro-environment elicits innate resistance to RAF inhibitors through HGF secretion. Nature.

[51] Mantovani A., Allavena P., Marchesi F., Garlanda C. (2022). Macrophages as tools and targets in cancer therapy. Nat. Rev. Drug. Discov..

[52] Kobayashi H., Enomoto A., Woods S.L., Burt A.D., Takahashi M., Worthley D.L. (2019). Cancer-associated fibroblasts in gastrointestinal cancer. Nat. Rev. Gastroenterol. Hepatol..

[53] Erin N., Grahovac J., Brozovic A., Efferth T. (2020). Tumor microenvironment and epithelial mesenchymal transition as targets to overcome tumor multidrug resistance. Drug. Resist. Updat..

[54] Mou Y., Wang J., Wu J., He D., Zhang C., Duan C., Li B. (2019). Ferroptosis, a new form of cell death: Opportunities and challenges in cancer. J. Hematol. Oncol..

[55] Du Y., Guo Z. (2022). Recent progress in ferroptosis: Inducers and inhibitors. Cell Death Discov..

[56] Yang W.S., SriRamaratnam R., Welsch M.E., Shimada K., Skouta R., Viswanathan V.S., Cheah J.H., Clemons P.A., Shamji A.F., Clish C.B. (2014). Regulation of ferroptotic cancer cell death by GPX4. Cell.

[57] Maiorino M., Conrad M., Ursini F. (2018). GPx4, Lipid Peroxidation, and Cell Death: Discoveries, Rediscoveries, and Open Issues. Antioxid. Redox Signal..

[58] Seiler A., Schneider M., Förster H., Roth S., Wirth E.K., Culmsee C., Plesnila N., Kremmer E., Rådmark O., Wurst W. (2008). Glutathione peroxidase 4 senses and translates oxidative stress into 12/15-lipoxygenase dependent- and AIF-mediated cell death. Cell Metab..

[59] Doll S., Proneth B., Tyurina Y.Y., Panzilius E., Kobayashi S., Ingold I., Irmler M., Beckers J., Aichler M., Walch A. (2017). ACSL4 dictates ferroptosis sensitivity by shaping cellular lipid composition. Nat. Chem. Biol..

[60] Yang W.S., Stockwell B.R. (2016). Ferroptosis: Death by Lipid Peroxidation. Trends Cell Biol..

[61] Bersuker K., Hendricks J.M., Li Z., Magtanong L., Ford B., Tang P.H., Roberts M.A., Tong B., Maimone T.J., Zoncu R. (2019). The CoQ oxidoreductase FSP1 acts parallel to GPX4 to inhibit ferroptosis. Nature.

[62] Dixon S.J., Lemberg K.M., Lamprecht M.R., Skouta R., Zaitsev E.M., Gleason C.E., Patel D.N., Bauer A.J., Cantley A.M., Yang W.S. (2012). Ferroptosis: An iron-dependent form of nonapoptotic cell death. Cell.

[63] Hentze M.W., Muckenthaler M.U., Galy B., Camaschella C. (2010). Two to tango: Regulation of Mammalian iron metabolism. Cell.

[64] Torti S.V., Torti F.M. (2013). Iron and cancer: More ore to be mined. Nat. Rev. Cancer.

[65] Wang D., Wu H., Yang J., Li M., Ling C., Gao Z., Lu H., Shen H., Tang Y. (2022). Loss of SLC46A1 decreases tumor iron content in hepatocellular carcinoma. Hepatol. Commun..

[66] Ohgami R.S., Campagna D.R., Greer E.L., Antiochos B., McDonald A., Chen J., Sharp J.J., Fujiwara Y., Barker J.E., Fleming M.D. (2005). Identification of a ferrireductase required for efficient transferrin-dependent iron uptake in erythroid cells. Nat. Genet..

[67] Stockwell B.R., Friedmann Angeli J.P., Bayir H., Bush A.I., Conrad M., Dixon S.J., Fulda S., Gascón S., Hatzios S.K., Kagan V.E. (2017). Ferroptosis: A Regulated Cell Death Nexus Linking Metabolism, Redox Biology, and Disease. Cell.

[68] Lee J., You J.H., Shin D., Roh J.-L. (2020). Inhibition of Glutaredoxin 5 predisposes Cisplatin-resistant Head and Neck Cancer Cells to Ferroptosis. Theranostics.

[69] Dixon S.J., Winter G.E., Musavi L.S., Lee E.D., Snijder B., Rebsamen M., Superti-Furga G., Stockwell B.R. (2015). Human Haploid Cell Genetics Reveals Roles for Lipid Metabolism Genes in Nonapoptotic Cell Death. ACS Chem. Biol..

[70] Rashba-Step J., Tatoyan A., Duncan R., Ann D., Pushpa-Rehka T.R., Sevanian A. (1997). Phospholipid peroxidation induces cytosolic phospholipase A2 activity: Membrane effects versus enzyme phosphorylation. Arch. Biochem. Biophys..

[71] Wenzel S.E., Tyurina Y.Y., Zhao J., St Croix C.M., Dar H.H., Mao G., Tyurin V.A., Anthonymuthu T.S., Kapralov A.A., Amoscato A.A. (2017). PEBP1 Wardens Ferroptosis by Enabling Lipoxygenase Generation of Lipid Death Signals. Cell.

[72] Ye L.F., Stockwell B.R. (2017). Transforming Lipoxygenases: PE-Specific Enzymes in Disguise. Cell.

[73] Zou Y., Li H., Graham E.T., Deik A.A., Eaton J.K., Wang W., Sandoval-Gomez G., Clish C.B., Doench J.G., Schreiber S.L. (2020). Cytochrome P450 oxidoreductase contributes to phospholipid peroxidation in ferroptosis. Nat. Chem. Biol..

[74] Shen F., Jiang G., Philips S., Gardner L., Xue G., Cantor E., Ly R.C., Osei W., Wu X., Dang C. (2023). Cytochrome P450 Oxidoreductase (POR) Associated with Severe Paclitaxel-Induced Peripheral Neuropathy in Patients of European Ancestry from ECOG-ACRIN E5103. Clin. Cancer Res. Off. J. Am. Assoc. Cancer Res..

[75] Huang N., Agrawal V., Giacomini K.M., Miller W.L. (2008). Genetics of P450 oxidoreductase: Sequence variation in 842 individuals of four ethnicities and activities of 15 missense mutations. Proc. Natl. Acad. Sci. USA.

[76] Liu M.-Y., Li H.-M., Wang X.-Y., Xia R., Li X., Ma Y.-J., Wang M., Zhang H.-S. (2022). TIGAR drives colorectal cancer ferroptosis resistance through ROS/AMPK/SCD1 pathway. Free. Radic. Biol. Med..

[77] Sun R., Lin Z., Wang X., Liu L., Huo M., Zhang R., Lin J., Xiao C., Li Y., Zhu W. (2022). AADAC protects colorectal cancer liver colonization from ferroptosis through SLC7A11-dependent inhibition of lipid peroxidation. J. Exp. Clin. Cancer Res..

[78] Shao Y., Jia H., Huang L., Li S., Wang C., Aikemu B., Yang G., Hong H., Yang X., Zhang S. (2021). An Original Ferroptosis-Related Gene Signature Effectively Predicts the Prognosis and Clinical Status for Colorectal Cancer Patients. Front. Oncol..

[79] Du S., Zeng F., Sun H., Liu Y., Han P., Zhang B., Xue W., Deng G., Yin M., Cui B. (2022). Prognostic and therapeutic significance of a novel ferroptosis related signature in colorectal cancer patients. Bioengineered.

[80] Sui X., Zhang R., Liu S., Duan T., Zhai L., Zhang M., Han X., Xiang Y., Huang X., Lin H. (2018). RSL3 Drives Ferroptosis Through GPX4 Inactivation and ROS Production in Colorectal Cancer. Front. Pharm..

[81] Han Y., Gao X., Wu N., Jin Y., Zhou H., Wang W., Liu H., Chu Y., Cao J., Jiang M. (2022). Long noncoding RNA LINC00239 inhibits ferroptosis in colorectal cancer by binding to Keap1 to stabilize Nrf2. Cell Death Dis..

[82] Yan H., Talty R., Jain A., Cai Y., Zheng J., Shen X., Muca E., Paty P.B., Bosenberg M.W., Khan S.A. (2023). Discovery of decreased ferroptosis in male colorectal cancer patients with KRAS mutations. Redox Biol..

[83] Wu T., Wan J., Qu X., Xia K., Wang F., Zhang Z., Yang M., Wu X., Gao R., Yuan X. (2023). Nodal promotes colorectal cancer survival and metastasis through regulating SCD1-mediated ferroptosis resistance. Cell Death Dis..

[84] Peng B., Peng J., Kang F., Zhang W., Peng E., He Q. (2022). Ferroptosis-Related Gene MT1G as a Novel Biomarker Correlated With Prognosis and Immune Infiltration in Colorectal Cancer. Front. Cell Dev. Biol..

[85] Wang R., Xing R., Su Q., Yin H., Wu D., Lv C., Yan Z. (2021). Knockdown of SFRS9 Inhibits Progression of Colorectal Cancer Through Triggering Ferroptosis Mediated by GPX4 Reduction. Front. Oncol..

[86] Xie Y., Zhu S., Song X., Sun X., Fan Y., Liu J., Zhong M., Yuan H., Zhang L., Billiar T.R. (2017). The Tumor Suppressor p53 Limits Ferroptosis by Blocking DPP4 Activity. Cell Rep..

[87] Yu Z., Tong S., Wang C., Wu Z., Ye Y., Wang S., Jiang K. (2022). PPy@Fe_3_O_4_ nanoparticles inhibit the proliferation and metastasis of CRC via suppressing the NF-κB signaling pathway and promoting ferroptosis. Front. Bioeng. Biotechnol..

[88] Hu Q., Wei W., Wu D., Huang F., Li M., Li W., Yin J., Peng Y., Lu Y., Zhao Q. (2022). Blockade of GCH1/BH4 Axis Activates Ferritinophagy to Mitigate the Resistance of Colorectal Cancer to Erastin-Induced Ferroptosis. Front. Cell Dev. Biol..

[89] Yang L., WenTao T., ZhiYuan Z., Qi L., YuXiang L., Peng Z., Ke L., XiaoNa J., YuZhi P., MeiLing J. (2022). Cullin-9/p53 mediates HNRNPC degradation to inhibit erastin-induced ferroptosis and is blocked by MDM2 inhibition in colorectal cancer. Oncogene.

[90] Fan H., Ai R., Mu S., Niu X., Guo Z., Liu L. (2022). MiR-19a suppresses ferroptosis of colorectal cancer cells by targeting IREB2. Bioengineered.

[91] Li Q., Li K., Guo Q., Yang T. (2023). CircRNA circSTIL inhibits ferroptosis in colorectal cancer via miR-431/SLC7A11 axis. Environ. Toxicol..

[92] Yang Y., Lin Z., Han Z., Wu Z., Hua J., Zhong R., Zhao R., Ran H., Qu K., Huang H. (2021). miR-539 activates the SAPK/JNK signaling pathway to promote ferropotosis in colorectal cancer by directly targeting TIPE. Cell Death Discov..

[93] Wei Z., Hang S., Wiredu Ocansey D.K., Zhang Z., Wang B., Zhang X., Mao F. (2023). Human umbilical cord mesenchymal stem cells derived exosome shuttling mir-129-5p attenuates inflammatory bowel disease by inhibiting ferroptosis. J. Nanobiotechnol..

[94] Tang F., Zhou L.-Y., Li P., Jiao L.-L., Chen K., Guo Y.-J., Ding X.-L., He S.-Y., Dong B., Xu R.-X. (2023). Inhibition of ACSL4 Alleviates Parkinsonism Phenotypes by Reduction of Lipid Reactive Oxygen Species. Neurotherapeutics.

[95] Zhang H., Deng T., Liu R., Ning T., Yang H., Liu D., Zhang Q., Lin D., Ge S., Bai M. (2020). CAF secreted miR-522 suppresses ferroptosis and promotes acquired chemo-resistance in gastric cancer. Mol. Cancer.

[96] Prevete N., Liotti F., Illiano A., Amoresano A., Pucci P., de Paulis A., Melillo R.M. (2017). Formyl peptide receptor 1 suppresses gastric cancer angiogenesis and growth by exploiting inflammation resolution pathways. Oncoimmunology.

[97] Du J., Wang X., Li Y., Ren X., Zhou Y., Hu W., Zhou C., Jing Q., Yang C., Wang L. (2021). DHA exhibits synergistic therapeutic efficacy with cisplatin to induce ferroptosis in pancreatic ductal adenocarcinoma via modulation of iron metabolism. Cell Death Dis..

[98] Chen Y., Yang Z., Wang S., Ma Q., Li L., Wu X., Guo Q., Tao L., Shen X. (2023). Boosting ROS-Mediated Lysosomal Membrane Permeabilization for Cancer Ferroptosis Therapy. Adv. Health Mater..

[99] Yu H., Yang C., Jian L., Guo S., Chen R., Li K., Qu F., Tao K., Fu Y., Luo F. (2019). Sulfasalazine-induced ferroptosis in breast cancer cells is reduced by the inhibitory effect of estrogen receptor on the transferrin receptor. Oncol. Rep..

[100] Hamad M., Mohammed A.K., Hachim M.Y., Mukhopadhy D., Khalique A., Laham A., Dhaiban S., Bajbouj K., Taneera J. (2021). Heme Oxygenase-1 (HMOX-1) and inhibitor of differentiation proteins (ID1, ID3) are key response mechanisms against iron-overload in pancreatic β-cells. Mol. Cell. Endocrinol..

[101] Sun Y., Qiao Y., Liu Y., Zhou J., Wang X., Zheng H., Xu Z., Zhang J., Zhou Y., Qian L. (2021). ent-Kaurane diterpenoids induce apoptosis and ferroptosis through targeting redox resetting to overcome cisplatin resistance. Redox Biol..

[102] Lin L., Shi Q., Su C.-Y., Shih C.C.Y., Lee K.-H. (2006). Antitumor agents 247. New 4-ethoxycarbonylethyl curcumin analogs as potential antiandrogenic agents. Bioorganic Med. Chem..

[103] Tu H., Tang L.J., Luo X.J., Ai K.L., Peng J. (2021). Insights into the novel function of system Xc- in regulated cell death. Eur. Rev. Med. Pharm. Sci..

[104] Zhang Q., Deng T., Zhang H., Zuo D., Zhu Q., Bai M., Liu R., Ning T., Zhang L., Yu Z. (2022). Adipocyte-Derived Exosomal MTTP Suppresses Ferroptosis and Promotes Chemoresistance in Colorectal Cancer. Adv. Sci..

[105] Chen H., Qi Q., Wu N., Wang Y., Feng Q., Jin R., Jiang L. (2022). Aspirin promotes RSL3-induced ferroptosis by suppressing mTOR/SREBP-1/SCD1-mediated lipogenesis in PIK3CA-mutatnt colorectal cancer. Redox Biol..

[106] Lin J.-F., Hu P.-S., Wang Y.-Y., Tan Y.-T., Yu K., Liao K., Wu Q.-N., Li T., Meng Q., Lin J.-Z. (2022). Phosphorylated NFS1 weakens oxaliplatin-based chemosensitivity of colorectal cancer by preventing PANoptosis. Signal. Transduct. Target. Ther..

[107] Yang C., Zhang Y., Lin S., Liu Y., Li W. (2021). Suppressing the KIF20A/NUAK1/Nrf2/GPX4 signaling pathway induces ferroptosis and enhances the sensitivity of colorectal cancer to oxaliplatin. Aging.

[108] Chaudhary N., Choudhary B.S., Shah S.G., Khapare N., Dwivedi N., Gaikwad A., Joshi N., Raichanna J., Basu S., Gurjar M. (2021). Lipocalin 2 expression promotes tumor progression and therapy resistance by inhibiting ferroptosis in colorectal cancer. Int. J. Cancer.

[109] Zeng K., Li W., Wang Y., Zhang Z., Zhang L., Zhang W., Xing Y., Zhou C. (2023). Inhibition of CDK1 Overcomes Oxaliplatin Resistance by Regulating ACSL4-mediated Ferroptosis in Colorectal Cancer. Adv. Sci..

[110] Zhou B., Mai Z., Ye Y., Song Y., Zhang M., Yang X., Xia W., Qiu X. (2022). The role of PYCR1 in inhibiting 5-fluorouracil-induced ferroptosis and apoptosis through SLC25A10 in colorectal cancer. Hum. Cell.

[111] Chen P., Li X., Zhang R., Liu S., Xiang Y., Zhang M., Chen X., Pan T., Yan L., Feng J. (2020). Combinative treatment of β-elemene and cetuximab is sensitive to KRAS mutant colorectal cancer cells by inducing ferroptosis and inhibiting epithelial-mesenchymal transformation. Theranostics.

[112] Hong T., Lei G., Chen X., Li H., Zhang X., Wu N., Zhao Y., Zhang Y., Wang J. (2021). PARP inhibition promotes ferroptosis via repressing SLC7A11 and synergizes with ferroptosis inducers in BRCA-proficient ovarian cancer. Redox Biol..

[113] Song X., Wang X., Liu Z., Yu Z. (2020). Role of GPX4-Mediated Ferroptosis in the Sensitivity of Triple Negative Breast Cancer Cells to Gefitinib. Front. Oncol..

[114] Fu D., Wang C., Yu L., Yu R. (2021). Induction of ferroptosis by ATF3 elevation alleviates cisplatin resistance in gastric cancer by restraining Nrf2/Keap1/xCT signaling. Cell. Mol. Biol. Lett..

[115] Zhang W., Luo M., Xiong B., Liu X. (2022). Upregulation of Metallothionein 1 G (MT1G) Negatively Regulates Ferroptosis in Clear Cell Renal Cell Carcinoma by Reducing Glutathione Consumption. J. Oncol..

[116] Sun X., Niu X., Chen R., He W., Chen D., Kang R., Tang D. (2016). Metallothionein-1G facilitates sorafenib resistance through inhibition of ferroptosis. Hepatology.

[117] Grignano E., Cantero-Aguilar L., Tuerdi Z., Chabane T., Vazquez R., Johnson N., Zerbit J., Decroocq J., Birsen R., Fontenay M. (2023). Dihydroartemisinin-induced ferroptosis in acute myeloid leukemia: Links to iron metabolism and metallothionein. Cell Death Discov..

[118] Gai C., Liu C., Wu X., Yu M., Zheng J., Zhang W., Lv S., Li W. (2020). MT1DP loaded by folate-modified liposomes sensitizes erastin-induced ferroptosis via regulating miR-365a-3p/NRF2 axis in non-small cell lung cancer cells. Cell Death Dis..

[119] Miyazaki I., Asanuma M. (2023). Multifunctional Metallothioneins as a Target for Neuroprotection in Parkinson’s Disease. Antioxidants.

[120] Yang J., Mo J., Dai J., Ye C., Cen W., Zheng X., Jiang L., Ye L. (2021). Cetuximab promotes RSL3-induced ferroptosis by suppressing the Nrf2/HO-1 signalling pathway in KRAS mutant colorectal cancer. Cell Death Dis..

[121] Tian X., Li S., Ge G. (2021). Apatinib Promotes Ferroptosis in Colorectal Cancer Cells by Targeting ELOVL6/ACSL4 Signaling. Cancer Manag. Res..

[122] Xia Y., Liu S., Li C., Ai Z., Shen W., Ren W., Yang X. (2020). Discovery of a novel ferroptosis inducer-talaroconvolutin A-killing colorectal cancer cells in vitro and in vivo. Cell Death Dis..

[123] Mu M., Zhang Q., Zhao C., Li X., Chen Z., Sun X., Yu J. (2023). 3-Bromopyruvate overcomes cetuximab resistance in human colorectal cancer cells by inducing autophagy-dependent ferroptosis. Cancer Gene Ther..

[124] Wang W., Green M., Choi J.E., Gijón M., Kennedy P.D., Johnson J.K., Liao P., Lang X., Kryczek I., Sell A. (2019). CD8+ T cells regulate tumour ferroptosis during cancer immunotherapy. Nature.

[125] Lei G., Zhuang L., Gan B. (2021). mTORC1 and ferroptosis: Regulatory mechanisms and therapeutic potential. Bioessays.

[126] Gao M., Monian P., Pan Q., Zhang W., Xiang J., Jiang X. (2016). Ferroptosis is an autophagic cell death process. Cell Res..

[127] Li Z., Chen L., Chen C., Zhou Y., Hu D., Yang J., Chen Y., Zhuo W., Mao M., Zhang X. (2020). Targeting ferroptosis in breast cancer. Biomark. Res..

[128] Wu Y., Zhang S., Gong X., Tam S., Xiao D., Liu S., Tao Y. (2020). The epigenetic regulators and metabolic changes in ferroptosis-associated cancer progression. Mol. Cancer.

[129] Lang X., Green M.D., Wang W., Yu J., Choi J.E., Jiang L., Liao P., Zhou J., Zhang Q., Dow A. (2019). Radiotherapy and Immunotherapy Promote Tumoral Lipid Oxidation and Ferroptosis via Synergistic Repression of SLC7A11. Cancer Discov..

[130] Zou Y., Schreiber S.L. (2020). Progress in Understanding Ferroptosis and Challenges in Its Targeting for Therapeutic Benefit. Cell Chem. Biol..

[131] Gu X., Liu Y.e., Dai X., Yang Y.-G., Zhang X. (2023). Deciphering the potential roles of ferroptosis in regulating tumor immunity and tumor immunotherapy. Front. Immunol..

[132] Shen D., Luo J., Chen L., Ma W., Mao X., Zhang Y., Zheng J., Wang Y., Wan J., Wang S. (2022). PARPi treatment enhances radiotherapy-induced ferroptosis and antitumor immune responses via the cGAS signaling pathway in colorectal cancer. Cancer Lett..

[133] Lv Y., Tang W., Xu Y., Chang W., Zhang Z., Lin Q., Ji M., Feng Q., He G., Xu J. (2023). Apolipoprotein L3 enhances CD8+ T cell antitumor immunity of colorectal cancer by promoting LDHA-mediated ferroptosis. Int. J. Biol. Sci..

[134] Wu J., Liu C., Wang T., Liu H., Wei B. (2023). Deubiquitinase inhibitor PR-619 potentiates colon cancer immunotherapy by inducing ferroptosis. Immunology.

[135] Chen C., Yang Y., Guo Y., He J., Chen Z., Qiu S., Zhang Y., Ding H., Pan J., Pan Y. (2023). CYP1B1 inhibits ferroptosis and induces anti-PD-1 resistance by degrading ACSL4 in colorectal cancer. Cell Death Dis..

[136] Shen Z., Song J., Yung B.C., Zhou Z., Wu A., Chen X. (2018). Emerging Strategies of Cancer Therapy Based on Ferroptosis. Adv. Mater..

[137] Qiao C., Wang H., Guan Q., Wei M., Li Z. (2022). Ferroptosis-based nano delivery systems targeted therapy for colorectal cancer: Insights and future perspectives. Asian J. Pharm. Sci..

